# The influence of feedstock characteristics on enzyme production in *Trichoderma reesei*: a review on productivity, gene regulation and secretion profiles

**DOI:** 10.1186/s13068-019-1571-z

**Published:** 2019-10-08

**Authors:** Vera Novy, Fredrik Nielsen, Bernhard Seiboth, Bernd Nidetzky

**Affiliations:** 1 Institute of Biotechnology and Biochemical Engineering, NAWI Graz, Graz University of Technology, Graz, Austria; 2 Institute of Chemical, Environmental and Bioscience Engineering, Vienna University of Technology, Vienna, Austria; 3 Austrian Centre of Industrial Biotechnology (acib) GmbH, Graz, Austria; 4 Present Address: Department of Wood Science, Faculty of Forestry, The University of British Columbia, Vancouver, Canada

**Keywords:** On-site enzyme manufacturing, Integrated enzyme manufacturing, *Trichoderma reesei*, Lignocellulose, Secretome, Transcriptome, Gene regulation, Productivity

## Abstract

Biorefineries, designed for the production of lignocellulose-based chemicals and fuels, are receiving increasing attention from the public, governments, and industries. A major obstacle for biorefineries to advance to commercial scale is the high cost of the enzymes required to derive the fermentable sugars from the feedstock used. As summarized in this review, techno-economic studies suggest co-localization and integration of enzyme manufacturing with the cellulosic biorefinery as the most promising alternative to alleviate this problem. Thus, cultivation of *Trichoderma reesei*, the principal producer of lignocellulolytic enzymes, on the lignocellulosic biomass processed on-site can reduce the cost of enzyme manufacturing. Further, due to a complex gene regulation machinery, the fungus can adjust the gene expression of the lignocellulolytic enzymes towards the characteristics of the feedstock, increasing the hydrolytic efficiency of the produced enzyme cocktail. Despite extensive research over decades, the underlying regulatory mechanisms are not fully elucidated. One aspect that has received relatively little attention in literature is the influence the characteristics of a lignocellulosic substrate, i.e., its chemical and physical composition, has on the produced enzyme mixture. Considering that the fungus is dependent on efficient enzymatic degradation of the lignocellulose for continuous supply of carbon and energy, a relationship between feedstock characteristics and secretome composition can be expected. The aim of this review was to systematically collect, appraise, and aggregate data and integrate results from studies analyzing enzyme production by *T. reesei* on insoluble cellulosic model substrates and lignocellulosic biomass. The results show that there is a direct effect of the substrate’s complexity (rated by structure, composition of the lignin–carbohydrate complex, and recalcitrance in enzymatic saccharification) on enzyme titers and the composition of specific activities in the secretome. It further shows that process-related factors, such as substrate loading and cultivation set-up, are direct targets for increasing enzyme yields. The literature on transcriptome and secretome composition further supports the proposed influence of substrate-related factors on the expression of lignocellulolytic enzymes. This review provides insights into the interrelation between the characteristics of the substrate and the enzyme production by *T. reesei*, which may help to advance integrated enzyme manufacturing of substrate-specific enzymes cocktails at scale.

## Background

### Enzyme production—a bottleneck in biochemical biorefinery processes

The circular economy and integrated biorefineries for valorization of lignocellulose have gained increased attention over the last decades [1]. The trend is driven by an increasing environmental awareness, as well as national and international policies and regulations for safer and more environmentally benign production processes [2]. Lignocellulosic biomass provides an abundant and relatively inexpensive raw material for biorefineries, and new technologies for the biochemical conversion of lignocellulose to value-added chemicals and fuels are emerging [1–3]. Applications thereby range from bulk to high-end products. However, there are inherent challenges in the biochemical conversion process that need to be addressed to be able to deploy these new technologies at scale.

A major challenge is the high cost of enzymes required to derive fermentable sugars from lignocellulose, especially for the biochemical conversion to bulk chemicals and fuels [4–7]. The required enzymes are a mix of cellulolytic, hemicellulolytic, and accessory proteins. They are typically purchased as standardized formulations from external suppliers and distributed from centralized production sites. However, it has become increasingly clear that standardized “one size fits all” formulations have limitations, as their performance varies in dependence of the type of biomass used in the conversion process [8–11]. The reason for this is that, depending on the feedstock used, a broad variety of different enzyme activities is required for efficient degradation (Table 1). Thus, there is a need to customize enzyme mixtures to maximize the hydrolytic efficiency and cost-effectiveness in the various conversion processes.

**Table 1:** Enzymes expressed and characterized in *T. reesei* for the degradation of hemicellulose and cellulose, grouped according to their functionality. Adapted from Häkkinen et al. [127]

Group	Functionality	Enzymes in *T. reesei*	EC
Enzymes for the degradation of hemicellulose			
Backbone cleaving enzymes	Degradation of the xylan backbone in arabinoxylan (hardwood) and arabinoglucoronoxylan (grasses) by endo- and exo-xylanases	Endo-β-1,4-xylanase	3.2.1.8
		1,4-β-Xylosidase	3.2.1.37
		Xyloglucan-specific endo-β-1,4-glucanase	3.2.1.151
	Degradation of the mannan backbone in galactoglucomannan (softwood) by endo- and exo-mannanases	Endo-1,4-β-mannosidase	3.2.1.78
		β-Mannosidase	3.2.1.25
		1,2-α-Mannosidase	3.2.1.113
		β-Galactosidase	3.2.1.23
Side-chain cleaving hydrolytic enzymes	Cleaving off galactose moieties from galactoglucomannan (softwood)	α-Galactosidase	3.2.1.22
	Cleaving off arabinose moieties from arabinoxylan (hardwood) and arabinoglucoronoxylan (grasses)	α-l-Arabinofuranosidase	3.2.1.55
	Cleaving off glucoronic moieties from arabinoxylan (hardwood) and arabinoglucoronoxylan (grasses)	α-Glucuronidase	3.2.1.139
Side chain cleaving esterases	Cleaving off acetyl groups from glucuronoxylan (hardwood), arabinoglucoronoxylan (grasses), and galactoglucomannan (softwood)	Acetyl xylan esterase	3.1.1.72
	Cleaving ester linkage between arabinose in hemicellulose and ferulic acid in lignin	Acetyl esterase	3.1.1.6
Enzymes for degradation of cellulose			
Concerted action of exo- and endo-cellulases and β-glucosidase		Endo-β-1,4-glucanase	3.2.1.4
		1,4-β-Cellobiosidase	3.2.1.91
		β-Glucosidase	3.2.1.21
Auxiliary activities	Cleavage of cellulose chains by oxidation of C1 or C4	Lytic polysaccharide monooxygenases	1.14.99.56
Non-hydrolytic proteins	High binding affinity for hemicellulose and cellulose, unknown role in biomass degradation	Swollenin	**–**

*Trichoderma reesei* is the principal producer of lignocellulolytic enzymes. The enzymes released into the culture supernatant are a mix of activities from different enzyme classes (Table 1). These activities act cooperatively in the degradation of lignocellulosic substrates. There is ample support from several studies for the following key assumptions of the analysis presented herein: (i) the composition of the enzyme mixtures determines the overall enzyme efficiency, (ii) different lignocellulosic substrates may require a different composition of the enzyme mixture for optimum degradation, due to variation in their chemical composition and structural/morphological characteristics, and (iii) cultivation of *T. reesei* on a lignocellulosic substrate results in an enzyme mixture adapted for degrading that particular substrate [9, 12–18]. By co-locating the enzyme production with the main biorefinery process, the lignocellulosic carbon source can be made accessible for fungal cultivations. In that way, a customized enzyme mixture may be produced through the efficient exploitation of natural mechanisms of adaptation. In addition, co-location has been a key assumption for achieving cost-competitiveness in several proposed lignocellulose-to-bioethanol processes [7, 19–22]. However, the regulatory machinery that allows the fungi to “sense” the substrate’s characteristics and adjust the gene expression pattern towards it is not fully understood.

As we will introduce hereinafter, there are several economic, environmental, and technical arguments to co-locate and integrate the enzyme production with lignocellulosic biorefineries. Literature review and meta-analysis further elucidate the interrelation between the physical and chemical composition of the substrate and the fungi’s secretome composition and the enzyme productivity. The presented insights may help to exploit *T. reesei* more efficiently for the on-site production of substrate specific enzyme mixtures at scale.

#### Economic aspects of on-site enzyme production

The high enzyme loadings required to deconstruct lignocellulosic biomass to fermentable sugars, in combination with the relatively low value of bulk products, necessitate cost-effective enzyme manufacturing. For many commoditized bulk chemicals and fuels, which compete on price with their petrochemical-based substitutes, enzymes may impose a prohibitive cost [4, 23]. One of the most studied cases is that of fuel ethanol from lignocellulosic feedstock. Several studies show that the cost of cellulolytic enzymes is a major contributor to the operating costs of cellulosic ethanol production [5, 6, 23–25]. The cost of enzymes is usually assessed by their cost contribution per produced volume of ethanol, and, in addition to the actual manufacturing costs, are heavily dependent on enzyme loading and overall ethanol yield [4]. A variation between 0.1 and 0.6 euro per liter of ethanol has been reported in Olofsson et al. [24] and references therein. In terms of cost contribution, enzymes are only superseded by that of the lignocellulosic raw material input [6, 23, 26]. Improving the enzyme productivity of the microorganisms, enhancing the hydrolytic capacity of the cellulases, and optimizing the technology of enzyme production are, therefore, essential to improve the cost-effectiveness of lignocellulose-based production processes [27]. The framework for enzyme production and cost optimization is segmented based on location and feedstock and can be divided into three scenarios: *off*-*site manufacturing*, *on*-*site manufacturing*, and the on-site subset *integrated manufacturing*.

In the *off*-*site manufacturing* scenario, cellulolytic enzyme preparations are manufactured by a large‐scale dedicated enzyme producer in a stand-alone plant. The enzyme production typically involves four main processes: (i) submerged cultivation of enzyme-producing microorganisms; (ii) separation and recovery of enzymes from the fermentation broth; (iii) concentration, preservation, and standardization of enzyme products; and (iv) inactivation of microorganisms and waste treatment. It has been suggested that off-site manufacturing could benefit from economies of scale [28] and have a near-term competitive advantage in optimized fermentation processes, achieving higher protein yields and cost-effectiveness [7, 28]. The capital investment is the main contributor to the cost of enzyme production in this scenario [4, 22]. The cost of raw materials further accounts for almost a third of the cost [4]. The respective contributions can be lowered by reducing the complexity of the enzyme recovery and formulation steps and shifting to lower-cost carbon and nitrogen sources [4]. However, best practice for commercial enzyme production and choice of carbon sources used are not publicly disclosed, which make estimates of production costs, and cost of raw materials in particular, highly uncertain [24, 27].

The alternative scenario is *on*-*site manufacturing* of enzymes with various degrees of process integration with the cellulosic biorefinery. In its most basic form, on-site manufacturing is a stand-alone production plant, equivalent to the off-site case, which is co-located with a cellulosic biorefinery. Co-location offers several compelling arguments regarding logistics, cost-effectiveness, and environmental impact. Cost reductions are achieved by sharing resources, e.g., land and buildings, waste treatment, and utilities infrastructure [7], thus reducing the capital investment contribution to the production cost. The proximity of the facilities minimizes the requirements for transportation and cold storage [7, 13]. The cellulolytic enzymes can be produced as whole broth that is directly used in enzymatic hydrolysis, thus avoiding costly cell removal, concentration, and formulation steps [13, 29]. Further improvement in cost-effectiveness can be achieved by integration of utilities (e.g., heat, cooling, and water) and process streams in the on-site manufacturing subset *integrated manufacturing* [7]. The anticipated most immediate cost benefits comes from shifting the primary carbon source to the lower-cost pretreated lignocellulose, bled from the biorefinery process streams [4, 13, 21, 23, 24]. Thus, existing infrastructure, logistics, and supply chains can be shared. Cost reductions of 5–25% have been proposed for on-site manufacturing of enzymes [23, 28], and optimistic projections of integrated manufacturing propose up to 70% reduction of operational costs [23]. However, any suggested potential for cost reduction is subject to significant uncertainties and numerous assumptions regarding the current state of enzyme production technology, production yields, choice of material inputs, scale of operation, and allocation of costs between functional units.

In addition to the cost reduction, using the on-site processed biomass for cultivations of *T. reesei* has the additional advantage that lignocellulose is a powerful inducing carbon source. It facilitates the gene expression of all enzyme classes required for the biomass’s saccharification, resulting in a strong hydrolytic potential of the cultivation supernatants [12–18]. Exploiting the regulatory mechanism of *T. reesei*, integrated enzyme manufacturing can enable the continuous adaption of the enzyme mixture to the feedstock at hand. This makes the biorefinery process more flexible with regard to choice of feedstock and, thus, results in a lower risk profile [3].

Life cycle analysis of stand-alone off-site manufacturing has shown that the contributions to greenhouse gas emissions are to a large extent driven by energy consumption, where fermentation and formulation of enzyme products are the main contributors [30]. Co-location and integration of enzyme manufacturing could lower those emissions by reducing heat requirements, shorten transportation distance, and avoid concentration and formulation steps [24, 28]. The potential for reduction of greenhouse gas emissions has been suggested to be in the range of 35–55% [24, 28].

On-site and integrated manufacturing strategies are embraced in proprietary cellulosic ethanol technologies, licensed by POET-DSM Advanced biofuels and Clariant. It is used at the POET-DSM commercial-scale plant in Emmetsburg (Iowa, USA) [31] and planned for the Clariant commercial-scale plant under construction in Podari (Romania) [32].

#### Insights into the regulatory network of *T. reesei*—an evolutionary optimized biomass degrader

*Trichoderma reesei* harbors complex regulatory mechanisms that enable it to fine-tune the expression and secretion of enzymes towards the substrate characteristics, an energy-conserving strategy for feedstock degradation. Gene expression of enzymes is mainly regulated at the transcriptional level, with the different classes of enzymes usually being co-regulated [33, 34] and their expression being dependent on the available carbon source. The rate of their transcription is controlled by a large set of transcription factors [35], and the most prominent ones are discussed below.

XYR1 is the master activator of cellulase gene expression [36] and it is necessary for the expression of cellulases and hemicellulases involved in xylan and arabinan degradation (Table 1) in the presence of inducing carbon sources [48–50]. Loss of XYR1 also affects the catabolism of lactose and different hemicellulose monomers, including d-xylose and l-arabinose [36–38]. An increase in *xyr1* transcript levels increases cellulase but not xylanase transcript levels [39]. Cellulase gene expression, as well as XYR1 expression itself, requires de novo biosynthesis of XYR1 and its simultaneous nuclear import [40].

In addition to XYR1, there are a number of other positive regulators described, including ACE2, ACE3, BglR, AZF1, VIB1, and the HAP2/3/5 complex. Deletion of *ace2* led to reduction of cellulase activity, specifically during growth on cellulose, but cellulase induction by sophorose was not affected [41]. Similarly, ACE3 is needed for high expression of cellulases and xylanases [42]. BglR was described as a positive regulator of β-glucosidases (excluding *bgl1*) [43]. Because its *Neurospora crassa* orthologue COL-26 was found to regulate both glucose sensing and glucose metabolism, BglR might have a broader function in regulation [44]. A loss of *AZF1* resulted in strongly reduced expression levels of cellulases [45]. VIB1, another regulator of cellulases, was found to be a functional homologue of the *N. crassa vib*-*1*. The latter is involved in the response to nitrogen and carbon starvation [46], and its deletion resulted in reduced cellulase expression [47]. Overexpression of *vib1* in *T. reesei* led to partially contradictory results as either no effect [47] or an increase in cellulase production was found [48]. The HAP2/3/5 complex binds the CCAAT box, a common motif in the eukaryotic promoter regions, and is involved in chromatin modification to activate gene expression [49].

Readily metabolizable carbon sources, including d-glucose and other monosaccharides, repress the expression of cellulases and xylanases. This effect, carbon catabolite repression (CCR), is mediated by CRE1 and enables *T. reesei* to adapt to changing carbon supplies, e.g., by preferentially using easily metabolizable sugar monomers over polysaccharides. CRE1 impairs cellulase production either indirectly, by repressing the expression of genes necessary for the uptake of inducers into the cell, or directly, by binding to the target genes [50]. A transcript analysis showed that only a limited number of CAZyme genes (a collection of all known and candidate Carbohydrate Active enZymes) are direct targets of CRE1 during CCR [50, 51]. Strains that either harbor a truncated version of *cre1* or have it deleted are derepressed for hemicellulose and cellulase expression. Under inducing conditions, these mutations further lead to an increased expression level [52], rendering *cre1* the prime target for creating enzyme hyperproducers. The industrial ancestor strain RUT-C30, for an instance, contains only a truncated *cre1* [52–55].

Other repressors are ACE1 and RCE1. The former, ACE1, represses cellulase and xylanase gene expression [56] and is itself subject to CRE1-dependent CCR [57]. Deletion of *rce1* resulted in a significant increase in extracellular cellulase activities on cellulose, but did not alter expression of xylanases during growth on xylan [58].

Chromatin represents another possibility for cellular regulation. Chromatin remodeling is necessary to promote cellulase expression and nucleosome rearrangements were found in the promoter regions of the major cellulases [49, 59]. A GCN5-like acetyltransferase, participating in the remodeling of chromatin by acetylating lysine residues in histones, is necessary for cellulase expression [60]. Another evidence for the role of chromatin comes from a study of the methyltransferase LAE1 [61]. Deletion and overexpression of *lae1* resulted in the impairment and promotion of cellulase expression, respectively, and is accompanied by changes in the H3K4 methylation pattern. The involvement of LAE1 and a second member of the velvet complex, VEL1 [62], in cellulase expression further indicates a cross-talk between fungal development and cellulase production.

Following the extracellular degradation of the lignocellulose, the uptake of the soluble breakdown products is a key process to regulate the transcription of cellulases and related genes. Here, the transporters play an important role, with some having the ability to sense the break-down products during their passage through the cell membrane. Two members of the MFS permease family, CRT1 and STP1, are involved in the regulation of cellulases. CRT1 was further speculated to partake in the cellulose sensing process [63, 64]. Another MFS transporter, STR1, is essential for pentose utilization and has been described to be involved in the induction of xylanase gene expression [65].

The precise mechanism by which carbon sources and other environmental signals regulate the expression of cellulases remains still unknown but within the last years, key regulators in different signal transduction pathways have been identified. The mitogen-activated protein kinases (MAPK) TMK1 and TMK2 repress cellulase formation, albeit not on the transcriptional level. In contrast TMK3, another MAPK, is directly involved in regulation of cellulase expression on the transcriptional level [66–68]. Deletion of an Ime2-like protein kinase not only led to an increase of cellulase induction in the early phase of growth on cellulose but also reduced the expression of *xyr1* and *cre1* [69].

Several studies have shown the involvement of light in the regulation of cellulase gene transcription, as reviewed here [70]. Important players are heterotrimeric G-proteins, the downstream cAMP pathway, as well as photoreceptors such as ENV1 and the blue light receptors BLR1 and BLR2 [70].

An important role was further ascribed to Ca^2+^, which affects the production and secretion of cellulases and xylanases, and can stimulate biomass growth [71]. A component of the Ca^2+^-responsive signaling pathway is the calcineurin-responsive zinc finger transcription factor CRZ1, which binds to the upstream regions of *xyr1* and *cbh1* and competes with the repressor ACE1 [71]. Similarly, Mn^2+^ stimulates cellulase production and protein secretion via calcium signaling. It regulates the calcium channels, which, in turn, leads to a significant increase in the cytosolic Ca^2+^ concentration. Excellent reviews of the current and combined knowledge of these regulatory systems have been published recently [70, 72–74].

#### Enzyme production by *T. reesei*—putting the substrate into the spotlight

In the integrated enzyme manufacturing scenario, *T. reesei* is cultivated on a lignocellulosic feedstock that has been treated by a commercially pursued pretreatment method, such as steam pretreatment [75]. On these feedstocks, fungal growth relies on the enzymatic hydrolysis of the structural carbohydrates in the biomass to sugar monomers and dimers. These sugars then serve as carbon and energy source, as well as inducers for continued enzyme production. Thus, the biomass growth and enzyme productivity of *T. reesei* is directly dependent on the efficiency of the enzymatic hydrolysis. As a consequence, it seems highly probable that the recalcitrance of the substrate is an influential factor in enzyme manufacturing. There is an abundance of data available in literature (as reviewed here [76–78]) that describe the impact of the lignocellulose characteristics on the efficiency of enzymatic hydrolysis. Studied factors include the ultrastructure of the cellulose, the accessibility of cellulose to cellulases, aspect ratio, pore size distribution, and the extent and nature of the ligno-carbohydrate complex (LCC), as well as the hemicellulose and lignin chemistry. Despite the extent of knowledge available from these enzyme-oriented studies, the structural features of the lignocellulosic substrates used for fungal cultivations receive relatively little attention in literature. As our systematic data collection shows (Additional file 1: Table S1), a multitude of studies lack description of feedstock treatment and basic biomass characterization, i.e., pretreatment conditions and chemical composition. To the best of our knowledge, there is currently no comprehensive study or literature-wide analysis which systematically evaluates the potential effects of the feedstock characteristics on the enzyme production by *T. reesei*. The aim of this study was, therefore, to systematically collect, aggregate, and appraise existing knowledge and analyze available data on the protein production by *T. reesei* cultivated on insoluble biomass.

## Meta-analysis of enzyme production by *T. reesei* cultivated on lignocellulosic substrates

To enable unbiased appraisal and evaluation of the influence of the substrate’s characteristics on the enzyme production, a systematic literature-wide search for original research papers (up until April 2019) was conducted. The data were collected and aggregated based on the inclusion criteria below.

The boundaries for the literature search was the following: (i) cultivation of *T. reesei* on insoluble substrates, i.e., cellulosic model substrates or complex agricultural or woody biomass; (ii) activity measurements in the secretome of at least one of the most commonly used enzyme assays, i.e., total cellulase activity on filter paper [79], protein concentration against a BSA standard [80], β-glucosidase activity on p-NPG [81], endoglucanase activity on carboxymethyl cellulose (CMC) [81], or xylanase activity on purified xylans. A summary of the studies that fit these criteria is given in Additional file 1: Table S1. We then used descriptive statistics and regression analysis to summarize the body of evidence from the included studies and to visualize our findings. Further, we used descriptive statistics as supporting evidence for the qualitative assessment of the included studies.

### The influence of media and process conditions on enzyme production

**Fig. 1:**
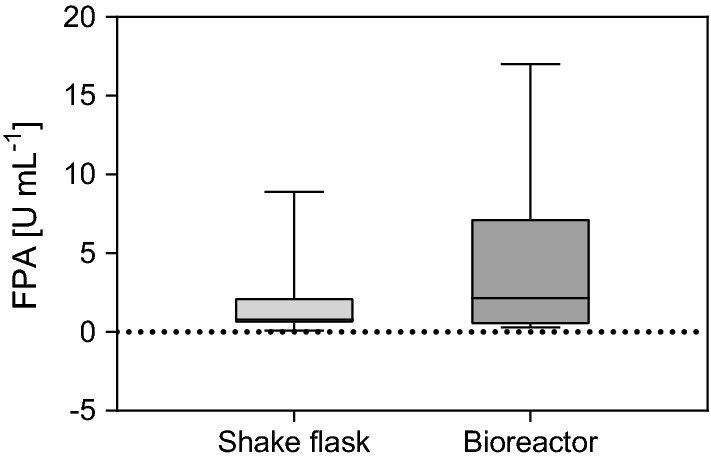
Box-and-whiskers plot for cellulase production in shake flask (*n* = 37) and bioreactor (*n* = 16) cultivations by *T. reesei*. Depicted are the min to max box plots in quartiles. The band inside the box represents the median

Although not directly connected to the substrate’s characteristics, the primary factors analyzed to optimize enzyme production in *T. reesei* have been the media and process conditions. Thus, studies have focused on optimizing the composition [13, 14, 82–88] as well as the pH [87, 89, 90] of the cultivation media. Because of the importance of mass, heat, and oxygen transport for fungal growth and enzyme productivity, detailed studies on the impact of aeration [85, 86, 91, 92] and agitation [85, 86, 91] were conducted. In the summary in Additional file 1: Table S1, we found that in 11 studies, bioreactors were used, and in 30 shake flasks. In contrast to shake flasks, bioreactors provide better mass, heat, and most importantly oxygen transfer, as well as stable and automatically regulated pH, temperature, and dissolved oxygen values. However, the stirrer, and the connected shear force, can have adverse effects on the hyphal biomass and enzyme productivity [91]. To dissect the potential bias due to variations in the process set-up, we analyzed if cultivations in bioreactors or shake flasks result in significant variations in FPA activity. The results are depicted in Fig. 1.

Independent of the carbon source, strain used, or other cultivation parameter, bioreactor cultivations result in higher total cellulase activities as compared to shake flask cultivations (Fig. 1). This is exemplified by the studies of Rodriguez-Gomez et al. and Bendig et al. who directly compared bioreactor and shake flask cultivations under else constant conditions. In all cases, the FPA titers achieved in bioreactor cultivations were higher than in shake flasks. In some cases, the improvements were several hundredfold [85, 86]. This suggests that the advantages of improved oxygen transfer and controlled pH, as reported earlier [85, 91–93], offsets the negative impact of the shear force on the hyphal biomass.

### The influence on the feedstock characteristics on enzyme productivities and titers

#### Assessing and categorizing lignocellulosic biomass

To aggregate information of the studies summarized in Additional file 1: Table S1, the substrates used for *T. reesei* cultivations were categorized based on their complexity. The complexity is an aggregate of (i) the degree of organization (ultrastructure), (ii) chemical composition of the lignin–carbohydrate complex (LCC), and (iii) its recalcitrance to deconstruction. The defined categories, sorted in ascending order of complexity, are: (1) Avicel, (2) Solka-Floc and pulp, (3) sugar cane bagasse, (4) herbaceous straw, and (5) woody biomass.

The first two categories are model cellulose substrates. The most commonly used and least complex substrate was microcrystalline cellulose, often referred to by the commercial name Avicel (*n* = 18) [48, 76, 82, 83, 85, 86, 88, 89, 91, 94–102]. It represents a highly pure and easy to mix cellulose powder with defined pore size distribution, aspect ratio, and crystallinity. It is virtually free of lignin and contains less than 3% hemicellulose (Additional file 1: Table S1). Solka-Floc and pulps are cellulose substrates with more of a fiber character than Avicel (*n* = 10) [14–16, 83, 84, 101, 103–106]. These are delignified substrates from various sources with a higher degree of polymerization than Avicel and contain up to 20% hemicellulose. The hemicellulose adds to the complexity of the substrate, and its content and type varies depending on source and treatment method.

The latter three categories are “real” substrates with relevance as feedstock in lignocellulosic biorefineries. These substrates typically need to be pretreated to disrupt the lignocellulosic matrix and render a larger fraction of the cellulose and hemicellulose accessible to the fungus. Despite its relevance for application, there is less data available on suitability of “real” substrates for enzyme production. Studies have investigated the use of sugar cane bagasse (*n* = 3) [88, 103, 107], herbaceous straw (wheat and rice straw, switch grass, corn stover, *n* = 10) [13, 15, 18, 76, 87, 99, 105, 108–110], and woody biomass (*n* = 4) [15, 90, 93, 105]. Dependent on pretreatment conditions, agricultural residues contain up to 25% hemicellulose, mainly xylan with few substituents. The lignin chemistry of herbaceous straws is reviewed elsewhere [111]. The coupling of xylan and lignin in the LCCs further increases the complexity and recalcitrance towards degradation [112, 113].

Lignin is regarded as a main source of biomass recalcitrance and low lignin content typically results in a better response to pretreatment and improved enzymatic digestibility. Higher lignin content, typically 20–25% in hardwoods and 25–30% in softwoods, differentiates woody biomass from the herbaceous straws used for biorefinery applications, and makes it more recalcitrant. Softwood is generally considered to be more recalcitrant than hardwoods. The difference is often attributed to the abundance of guaiacyl units in softwood lignin, which are more prone to repolymerize and form recalcitrant structures during pretreatment than syringyl units (predominant in hardwoods) [114]. Molecular-level structures and functional groups on the lignin polymer also contribute to its recalcitrance [114].

Please note, the categories presented herein are based on typical substrate characteristics. By selecting pretreatment method and conditions, substrate properties such as accessibility, hydrolyzability, hemicellulose and lignin content, particle size, and porosity can be manipulated [115, 116]. Thus, the substrate characteristics are dependent on biomass type and source, as well as treatment method. Severe pretreatment conditions thereby can lead to secondary decomposition processes and the formation of inhibitory compounds, such as acetic acid and furaldehydes [115, 116]. These compounds can have negative effects on the enzyme productivity and viability of the fungus [88, 90, 117] as well as on the enzyme–substrate interaction [77, 118, 119]. Although it will be important to tailor pretreatment and substrate preparation to accommodate both enzyme production and downstream processing in any on-site scenario (also see “Economic aspects of on-site enzyme production”), this aspect is beyond the scope of the present review and will not be discussed in more detail hereinafter.

#### Influence of the feedstock complexity on enzyme production

**Fig. 2:**
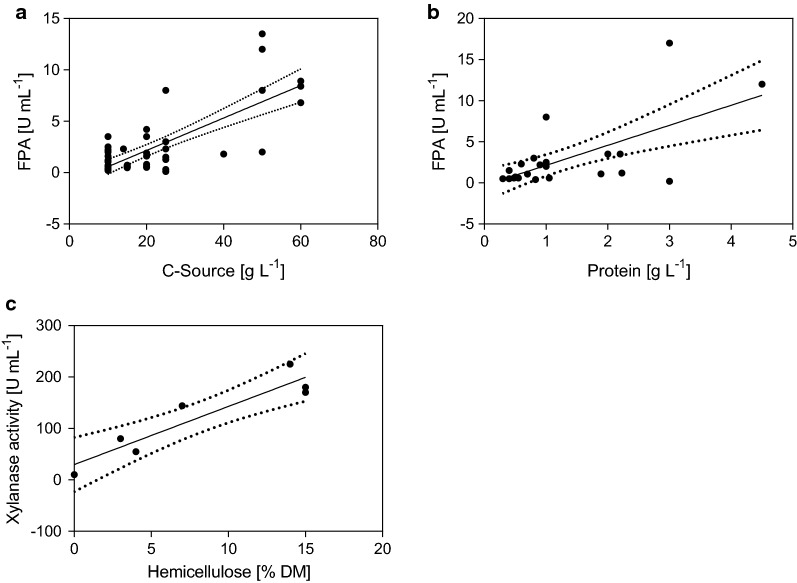
Metadata analysis on enzyme production by *T. reesei* on insoluble substrates. Depicted is the correlation between the substrate concentration and the FPA (**a**), the protein concentration and the FPA (**b**), and the hemicellulose content of the substrate and the xylanase activity (**c**). Data are summarized in Additional file 1: Table S1. The solid line represents the linear regression of the data points, the dotted line the 95% confidence interval

**Fig. 3:**
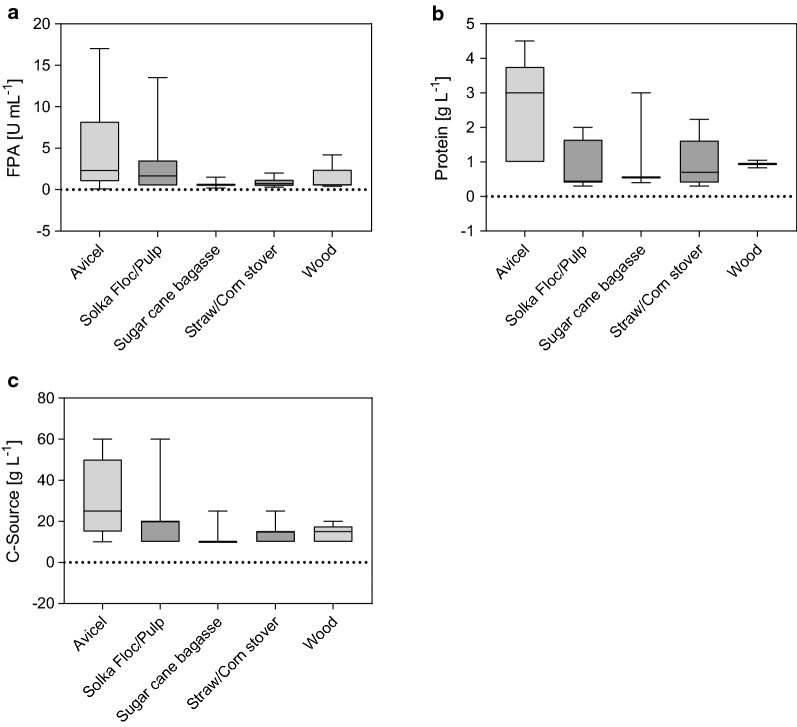
The influence of substrate type on FPA production (**a**), protein production (**b**), and C-source concentration utilized (**c**). The categories were ordered with ascending complexities from left to right, where the complexity is a function of the structural organization, the chemical composition, and the recalcitrance to deconstruction (as detailed in “Assessing and categorizing lignocellulosic biomass” section). Depicted are the min to max box plots in quartiles. The band inside the box represents the median. The raw data with the respective references can be found in Additional file 1: Table S1

The influence of the substrate complexity on total (FPA) and single (xylanase, β-glucosidase and endoglucanase) enzyme activities in the in *T. reesei*’s secretome of studies summarized in Additional file 1: Table S1 was analyzed. The results are depicted in Figs. 2 and 3. In contrast to the comparison of the cultivation set-ups (i.e., bioreactor vs shake flask, also see “The influence of media and process conditions on enzyme production” section), only data from carbon catabolite derepressed strains (i.e., RUT-C30 or comparable *cre1* mutant strains) were included from Additional file 1: Table S1, to facilitate a fair comparison.

As depicted in Fig. 2, reported total cellulase activities (FPA) vary from below 1 to almost 15 U mL^−1^, and seemingly depend on the substrate concentration (Fig. 2a), with the indicated regressed linear correlation having a slope of 0.16 (*R*^2^ 0.56). Kinetic studies of *T. reesei* on insoluble substrates have shown that cellulase production is following three phases; (i) a lag phase (high biomass growth, low cellulase productivity); (ii) a cellulase production phase (low biomass growth, high cellulase productivity), and (iii) an end phase (no biomass production, decreasing cellulase productivity) initiated with the depletion of the substrate [96, 106]. Thus, at higher substrate concentrations the phase in which enzyme production is continuously induced is longer, resulting in higher FPA titers (Fig. 2a). However, due to the adverse effect of the insoluble substrates on the rheology of the cultivation broth, there is a substrate-specific threshold value, above which limitation to the mass and heat transfer occurs [13, 91, 92, 96, 101].

When plotting reported FPA against the respective protein concentrations, a weak correlation can be detected (Fig. 2b). The slope of the indicated regressed linear correlation is 2.44 (*R*^2^ 0.42), suggesting an overall specific cellulase activity of approximately 2.4 FPA per mg protein.

We further investigated the distribution of other, routinely measured enzyme activities. No correlation to any of the investigated parameters was found in case of the endoglucanase (EG) activity or the β-glucosidase (BGL) activity (Additional file 1: Table S1). However, reported xylanase activities were especially high in studies on complex lignocellulosic substrates [13, 15, 17, 100, 104]. Hassan et al. [120] described the increase in produced xylanase activities in cultivations of *T. reesei* RUT C-30 on three cellulosic substrates with increasing hemicellulose content. Even a small increase in the hemicellulose content was detectable in the secretome. Studies that conducted fungal cultivations on the same feedstock pretreated under varying conditions, further described a decrease in xylanase activity with increasing pretreatment severities [90, 121]. Higher pretreatment severities are usually accompanied by an increasing loss in hemicellulose content, due to solubilization and secondary degradation processes [75].

To investigate the potential relationship between the substrate’s hemicellulose content and the xylanase activity in more detail, we extracted data from Additional file 1: Table S1 in which (i) a complex lignocellulosic substrate containing hemicellulose was utilized, (ii) the compositional analysis of the substrate was given, and (iii) a comparable xylanase assay, i.e., on pure xylan, was performed. Of all studies in Additional file 1: Table S1, seven cases fulfilled these requirements. As depicted in Fig. 2c, the produced xylanase activity showed a strong positive correlation with the hemicellulose content (the slope of regressed linear correlation is 11.3 with a *R*^2^ of 0.86). This suggests that the presence of hemicellulose in the material is required for the induction of hemicellulase production.

In the next step, the reported enzyme titers were categorized according to the complexity of the substrate the fungus was cultivated on, representing the five different groups introduced in “Assessing and categorizing lignocellulosic biomass” section. The results are summarized in Fig. 3. An overall trend of decreasing cellulase titers with increasing substrate complexity can be observed, where cultivation on Avicel generally yielded the highest titers (Fig. 3a). It lacks lignin and only contains traces of hemicellulose (“Assessing and categorizing lignocellulosic biomass” section), the accessibility for enzymes to cellulose is, therefore higher in Avicel than in the other substrates [76–78]. Because the induction of gene expression as well as fungal growth is dependent on a continuous release of inducing sugars, substrates with a high accessibility and hydrolyzability are preferred. Further, the degradation of pure cellulosic substrates mainly requires the exo- and endo-acting cellulases (CBHI and II, EGs, Table 1) in coordination with BGLs.

Finally, Peciulyte et al. [101] have shown that cultivation on Avicel results in up to sixfold higher FPA titers as compared to other cellulosic substrates, e.g., pulps. The authors attributed this effect, amongst other factors, to the lower viscosity of the Avicel as compared to the pulp containing cultivation broths, resulting in better mass, heat, and oxygen transfer. This would also imply that more substrate can be loaded while maintaining sufficient mass, heat, and oxygen transfer. This is supported by our analysis (Fig. 3c), where the lower complexity substrates were loaded at higher concentrations on average than their higher complexity counterparts.

When comparing the “real” lignocellulosic substrates, woody biomasses seem to result in higher enzyme production than herbaceous straws (Fig. 3). Although dependent on wood source, i.e., soft- or hardwood, woody biomass in general is more difficult to hydrolyze than its agricultural counterparts [76, 78]. The explanation for this phenomenon might lay in the fact that the induction of the full array of cellulases, hemicellulases, and accessory proteins (Table 1) require specific, not always known, inducing compounds (also see “Complex lignocellulosic substrates—the expression of hemicellulases, and accessory proteins and enzymes” section). If present in the feedstock used for cultivation, these enzymes get secreted by the fungus, rendering the enzyme cocktail more powerful in the degradation of the biomass at hand [13–18]. Thus, substrates that are commonly perceived as highly recalcitrant, due to the slow or incomplete hydrolysis by standardized commercial enzyme cocktails, might be more efficiently degraded by the tailored fungal enzyme mixture [9].

In summary, more complex substrates result not only in lower production but also in a more diverse set of enzymes produced (Additional file 1: Table S1). Due to the intertwined nature of process conditions, fungal growth, and enzyme productivity, improvements might be achieved by avoiding highly viscous media, e.g., by size reduction or in fed-batch approaches.

## Effect of the substrate characteristics on the fungal transcriptome and secretome

The complete deconstruction of the carbohydrate polymers in lignocellulose requires a diverse set of different enzyme activities. An overview of described and characterized hemicellulolytic and cellulolytic enzymes in *T. reesei* is given in Table 1. To investigate the pattern behind the regulation of gene expression of these enzyme classes, studies conducted genome-wide analyses of the fungal transcriptome using several different techniques. These included RNA sequencing [50, 104, 122–125], microarray analysis [42, 126–129], and quantitative PCR [123, 130–132]. The protein abundance in the secretome was further analyzed and quantified by mass spectrometry [94, 101, 125, 131–135].

The following section focuses on studies on analyzing the fungal transcriptomes and secretomes using insoluble cellulose model and “real” lignocellulosic substrates. These included Avicel [50, 94, 101, 120, 123, 125, 132, 133, 136], Solka-Floc [33, 104, 130, 137], wheat straw [122, 127, 128], corn stover [133], sugar cane bagasse [126, 127, 131] and various hemicellulose-derived materials [137, 138].

### Soluble vs simple cellulosic substrates—the expression of cellulases

To understand the gene regulation behind carbon catabolite repression, studies investigated the fungal transcriptome and secretome under inducing, non-inducing or repressing conditions [33, 34, 50, 51, 94, 123, 125, 137, 138]. As inducing carbon source, sugars, e.g., lactose and sophorose, or model cellulosic substrates, e.g., Avicel, were used [33, 42, 94, 125, 127, 130, 137, 138]. Lactose is considered to be recognized as an inducer by the fungus because it resembles the hydrolyzed β-galactoside side chains of xyloglucans [64]. Sophorose, a powerful inducer of cellulases, is a transglycosylation product of cellobiose by BGL [33, 102, 139]. Recent studies have shown that the transglycosylation activity of BGL can be exploited to generate artificial inducers from glucose, improving enzyme titers up to 17-fold [140, 141].

Collectively, these studies have resulted in the ability to generate *T. reesei* mutant strains that are carbon catabolite derepressed, realizing higher enzyme titers. These strains are enabled to produce cellulase and hemicellulases on carbon sources that would lead to complete or partial repression in wild type strains [50–52, 55, 102, 108, 110, 142–146].

Despite this, Ilmén et al. [33] demonstrated that induction of the “classic enzymes” (CBH I and II, EG 1–5, Table 1) in cultivations on Solka-Floc is superior to the soluble inducing substrates cellobiose and lactose. Studies further demonstrated that the composition of the transcriptome and secretome varies between the simple inducing sugars cellobiose and sophorose, and cellulosic substrates [94, 123, 125, 130], although they are all degradation products of cellulose. *T. reesei* lacking the *cre1* transcription factor further showed increased induction of cellulase gene expression on cellulose, but not on glucose. During growth on glucose, cellulase transcripts appeared only after prolonged incubation and were generally lower. This was taken as evidence that, irrespective of the mechanism behind CCR, the fungus can distinguish if glucose was provided as sugar monomer or released from cellulose [51, 52, 94]. Variation in the secretome composition was even detected between cellulosic substrates that almost exclusively vary in their ultrastructure [101]. These studies are evidence that even on the homogenous polymer cellulose, gene expression is regulated by a vast and complex machinery, including many, currently unknown, substrate-related factors (see “Insights into the regulatory network of *T. reesei*—an evolutionary optimized biomass degrader” section).

It was further shown that the genes encoding CBH and EG enzymes are co-regulated [34, 36, 42, 127, 147]. Cellulose hydrolysis relies on the exo–endo synergism of these two enzyme classes, rendering this co-regulation important for efficient feedstock degradation. Alongside the CBHs and EGs, cellulose induces the expression of the non-hydrolytic protein swollenin (SWO1). Although no clear role of SWO1 in cellulose hydrolysis was found so far, its overexpression on cellulosic substrates has been described in many studies [122, 126, 127, 129, 130]. Considering the small genome of *T. reesei* [148] that has evolved to be highly efficient and energy-conserving [104], it is unlikely that a protein like swollenin is secreted by the fungus without benefits for it.

In addition to the cellulases, cellulose further resulted in the upregulation of a transporter (MFS permease) and the β-mannanase MAN1 [94, 125] gene. The transporter likely plays a role in nutrient signaling ([63, 64, 128, 132] and “Insights into the regulatory network of *T. reesei*—an evolutionary optimized biomass degrader” section). β-Mannanase is mainly required for the degradation of galactoglucomannans in softwood (Table 1), and this finding suggests that pure cellulose not only induces the cellulases but also enzymes with hemicellulolytic activities.

### Complex lignocellulosic substrates—the expression of hemicellulases, and accessory proteins and enzymes

Margolles-Clark et al. [137] investigated the transcription profiles of cellulolytic and hemicellulolytic enzymes. Similar to the findings of Dos Santos Castro et al. [94, 125], many of the backbone and side-chain cleaving hemicellulolytic enzymes (Table 1) were induced by the hemicellulose containing substrates as well as on pure cellulose. Considering the tight association in native lignocellulosic feedstock (see “Assessing and categorizing lignocellulosic biomass” section), co-expression of hemicellulases and cellulases is required for efficient feedstock degradation. However, the authors also found that complex substrates (e.g., oat spelt) induce the expression of a broader array of genes, even when compared to their “cleaner” counterparts (e.g., purified xylan). Adav et al. [133] compared the secretomes of fungal cultivations on cellulose, corn stover, and saw dust. They identified 230 proteins, including cellulose, hemicellulose, and lignin degrading enzymes, in the secretomes and quantified them. They found that the secretome profiles vary significantly between the carbon sources. Thus, going from pure cellulose to saw dust and corn stover an increase in abundancy of all cellulases and a broader variety of hemicellulolytic enzyme activities was detected. Although not specified, it might be that in corn stover, the hemicellulose was more readily accessible and contained a different heteropolymer composition than saw dust. A similar picture was presented by Bischof et al. and Ries et al. [122, 128] who analyzed the fungal transcriptome on wheat straw. In the former study, the transcriptome of *T. reesei* was compared to that acquired on lactose. The authors found that although lactose induces ~ 60% of the CAZyme genes; the level of upregulation was weaker as compared to wheat straw. Lactose further does not, or only mildly, induce specific xylan- and arabinan-degrading enzymes (Table 1). Supported by earlier studies [37, 143], it was concluded that the induction of gene expression of these enzymes requires the presence of the specific lignocellulose-derived inducers. Similar to Adav et al. [133], an increase in chitinases, α-galactosidases and mannosidases gene expression was detected [128]. Adav et al. [133] related the strong upregulation of gene expression of mannosidases to the ability of *T. reesei* to grow on softwood (Table 1). In contrast, Bischof et al. [128] suggested that they are expressed due to a state of starvation, and the resulting onset of autophagy.

In the study of Häkkinen et al. [127], a wide variety of substrates was used to analyze the impact of the substrate composition on the fungal transcriptome. Cluster analysis showed that the enzyme group, the genes of which are induced the strongest on hemicellulose containing substrates (steam pretreated bagasse and wheat straw) encompassed most of the known and candidate hemicellulases. This provides conclusive evidence that hemicellulose chemistry directly influences the fungal transcriptome. The authors further suggested that the nature of the side chains (Table 1) plays a role in the induction process [127]. Apart from the substrate-dependent variations in the transcriptome profiles, Häkkinen et al. [127] described a time-dependent change of it. Because cellulose is embedded in a matrix of various hemicellulose polymers and lignin, a cascaded secretion of different enzyme activities, that can sequentially deconstruct the hemicellulose and lignin shields, can increase the hydrolysis efficiency.

Transcriptome analysis was also conducted in cultivations on sugar cane bagasse [126, 131], with largely similar trends as discussed above. In the work of Borin et al. [126], the transcriptome additionally detected the upregulation of LPMO (lytic polysaccharide monooxygenase) gene expression. LPMOs are oxidative enzymes that can attack cellulose but require an electron donor [149]. It has been suggested that lignin can provide the electrons required [150]. Interestingly, the LPMOs in *T. reesei* seem to be co-regulated with alcohol oxidases, aryl-alcohol oxidases, and glucose oxidases [126]. Because these enzymes form hydrogen peroxide and oxygen radicals, they might be responsible to oxidize phenolic compounds in the lignin while reducing the LPMO [126, 149]. The proposed mechanism has so far not been described in other studies, but elucidation of the mechanism could lead the way towards better understanding of how *T. reesei* can decompose lignocellulosic substrates.

## Conclusion

Collective information from techno-economic analyses show that shifting from off-site to on-site and integrated enzyme manufacturing can cut production costs by up to 70%. By collecting data of enzyme titers, total cellulase and single enzyme activities in the secretome, parameters with a pronounced impact on enzyme productivity could be dissected. Thus, controlling the cultivation conditions (i.e., oxygen, temperature, and pH) can increase the average enzyme titer significantly, with reported enzyme titers varying from 0.1 to 8.0 FPU mL^−1^ in shaken flaks and 0.5 to 17.0 FPU mL^−1^ in bioreactors. Further, a strong positive correlation between substrate concentration (10–60 g L^−1^) and cellulase activity (0.1–17 FPU mL^−1^) was observed. The hemicellulose content (0.1–17% dry matter) of the substrate used positively correlated with the reported xylanase activity (10–225 U mL^−1^). Data categorized according to the complexity of the substrate used (rated by structure, chemical composition, and recalcitrance) showed that Avicel generally yielded the highest enzyme titers, followed by cultivations conducted on pulp, wood, herbaceous straws, and sugar cane bagasse in a declining trend (Fig. 3a). More detailed insights of the impact of the substrate on gene regulation were gained by a literature review of transcriptome and secretome studies. Here, the cellulose structure, the hemicellulose chemistry, i.e., backbone and side-chain composition, and the lignin content were described to directly affect gene regulation in *T. reesei*. Thus, specific hemicellulose-derived inducers are required to upregulate the full array of hemicellulolytic enzymes. This implies that cultivation of the fungus on a substrate with a complex hemicellulose composition (e.g., the galactoglucomannan of softwood) will result in induction of the related-enzyme classes (e.g., mannanases, galactosidases). In turn, the cultivation supernatant will excel in the saccharification of that precise feedstock.

This systematic review suggests that the substrate characteristics are directly affecting enzyme titers and secretome compositions in cultivations of *T. reesei*, resulting in an enzyme cocktail that is optimized for that precise biomass. In view of process integration of enzyme manufacturing and cellulosic biorefineries, exploiting the fungi’s substrate “sensing” can be a key to produce efficient tailored enzyme cocktails in an economically viable and greener way.

## Supplementary information

**Additional file 1.** Collection of data from the systematic literature-wide search that fitted the inclusion criteria set in this study (please refer to the “Meta-analysis of enzyme production by *T. reesei* cultivated on lignocellulosic substrates” section).

## Data Availability

All data generated or analyzed during this study are included in this published article and its Additional file.

## Abbreviations

BGL: β-glucosidase
CBH: cellobiohydrolase
CCR: carbon catabolite repression
CMC: carboxymethyl cellulose
EG: endoglucanase
FPA: filter paper activity
LPMO: lytic polysaccharide monooxygenase
LCC: ligno-carbohydrate complex
MAPK: mitogen-activated protein kinases
